# Decreased mTOR signaling pathway in human idiopathic autism and in rats exposed to valproic acid

**DOI:** 10.1186/s40478-015-0184-4

**Published:** 2015-01-20

**Authors:** Chiara Nicolini, Younghee Ahn, Bernadeta Michalski, Jong M Rho, Margaret Fahnestock

**Affiliations:** Department of Psychiatry & Behavioural Neurosciences, McMaster University, 1280 Main Street West, Hamilton, ON L8S 4K1 Canada; Departments of Paediatrics & Clinical Neurosciences, Alberta Children’s Hospital Research Institute, University of Calgary, Calgary, AB T3B 6A8 Canada

**Keywords:** Human postmortem, Autism, Valproate, Signal transduction, TrkB, PSD-95

## Abstract

**Background:**

The molecular mechanisms underlying autistic behaviors remain to be elucidated. Mutations in genes linked to autism adversely affect molecules regulating dendritic spine formation, function and plasticity, and some increase the mammalian target of rapamycin, mTOR, a regulator of protein synthesis at spines. Here, we investigated whether the Akt/mTOR pathway is disrupted in idiopathic autism and in rats exposed to valproic acid, an animal model exhibiting autistic-like behavior.

**Methods:**

Components of the mTOR pathway were assayed by Western blotting in postmortem fusiform gyrus samples from 11 subjects with idiopathic autism and 13 controls and in valproic acid versus saline-exposed rat neocortex. Additionally, protein levels of brain-derived neurotrophic factor receptor (TrkB) isoforms and the postsynaptic organizing molecule PSD-95 were measured in autistic versus control subjects.

**Results:**

Full-length TrkB, PI3K, Akt, phosphorylated and total mTOR, p70S6 kinase, eIF4B and PSD-95 were reduced in autistic versus control fusiform gyrus. Similarly, phosphorylated and total Akt, mTOR and 4E-BP1 and phosphorylated S6 protein were decreased in valproic acid- versus saline-exposed rats. However, no changes in 4E-BP1 or eIF4E were found in autistic brains.

**Conclusions:**

In contrast to some monogenic disorders with high rates of autism, our data demonstrate down-regulation of the Akt/mTOR pathway, specifically via p70S6K/eIF4B, in idiopathic autism. These findings suggest that disruption of this pathway in either direction is widespread in autism and can have adverse consequences for synaptic function. The use of valproic acid, a histone deacetylase inhibitor, in rats successfully modeled these changes, implicating an epigenetic mechanism in these pathway disruptions.

## Introduction

Autism is a neurodevelopmental disorder characterized by social communication and interaction impairments and restricted, repetitive patterns of behavior [1]. While it is believed that defects in the establishment and maintenance of functional neuronal networks due to synaptic/spine dysfunction underlie the clinical symptomatology of autism, the molecular mechanisms causing these defects remain unknown. Several studies point to the Akt/mTOR pathway, which regulates translation at dendritic spines [2,3], as a potential molecular substrate of autism. Indeed, mutations in genes encoding Akt-mTOR cascade components cause disorders with high rates of autism [2-5]. Additionally, autism-like phenotypes have been observed in Eif4ebp2 knockout and eIF4E-overexpressing mice [6], both downstream mTOR effectors regulating protein translation. These findings strengthen the hypothesis that disruptions in the mTOR pathway contribute to autism neuropathology, likely by negatively affecting spines, which are perturbed in subjects with autism [7]. Nonetheless, protein products of these genes have not been examined in idiopathic autism.

Valproic acid (VPA) is widely used as an antiepileptic drug [8] and for the treatment of mood disorders [9,10]. Maternal exposure to VPA at the time of neural tube closure causes autism-like symptoms in humans [11-13]. Clinical effects of maternal challenge with VPA, including impaired social interactions, stereotypical hyperactivity and sensory/communication deficits have been reproduced in both rats and mice [14-17]. In particular, prenatally VPA-exposed rats exhibit not only autistic-like behaviors [18,19] but also anatomical and molecular alterations similar to human autism including reductions in cerebellar volume and numbers of Purkinje cells [20,21], altered monoamine levels [22], decreased expression of the autism-linked gene, neuroligin 3 [23], and perturbed cortical connectivity along with abnormal synapse formation and pruning [24,25]. VPA’s ability to inhibit histone deacetylase (HDAC) has been associated with autistic-like behavioural, anatomical and biochemical deficits in mice prenatally exposed to this drug [17], supporting the hypothesis that epigenetic mechanisms contribute to autism etiology. Indeed, mounting evidence highlights the involvement of epigenetic factors in synaptic dysfunction and disrupted cortical circuitry causing autistic traits [26,27]. However, the affected signal transduction pathways remain to be identified.

In the present study, we investigated whether the Akt/mTOR pathway, which is disrupted in monogenic disorders with high rates of autism, is altered in idiopathic autism. We specifically examined idiopathic autism, that is, subjects without known genetic causes of autism and without related disorders on the spectrum. We also examined whether VPA exposure in rats affects this pathway. We measured protein levels of mTOR and its upstream/downstream effectors in brains from subjects with idiopathic autism versus control subjects and in VPA- versus saline-exposed rats. Contrary to disruptions seen in Fragile X syndrome, tuberous sclerosis, PTEN-related macrocephaly and neurofibromatosis type 1, developmental disorders with high rates of autism, we demonstrated decreased Akt/mTOR pathway in both patients with idiopathic autism and in VPA-exposed rats. These findings support the hypothesis that alterations in Akt/mTOR signaling in either direction contribute to autistic behavior, likely by affecting dendritic spines. Additionally, similar molecular changes in VPA-exposed rats suggest that epigenetic changes may underlie the synaptic deficits characteristic of idiopathic autism.

## Materials and methods

### Human brain tissue samples

All experimental protocols were approved by the Research Ethics Board of McMaster University. Frozen samples consisting of eleven post-mortem fusiform gyrus samples from subjects with idiopathic autism (3 females, 8 males) and thirteen control brain samples (3 females, 10 males) were provided to us by the Autism Speaks’ Autism Tissue Program (Princeton, NJ) via the Harvard Brain Tissue Resource Centre (Belmont, MA) and the National Institute of Child Health and Human Development (NICHD) Brain and Tissue Bank (University of Maryland, Baltimore, MD) and were stored at −80°C before use. Clinical information about each tissue sample was obtained through the Autism Tissue Program online portal [http://www.autismbrainnet.com/about-us/portal/] [28] (Tables 1 and 2). Samples were matched as much as possible for age, gender and post-mortem interval (PMI). There were no significant differences between groups for these variables [28]. Cause of death, however, differed between samples. It was not possible to match the cause of death due to the scarcity of available tissue. All of the tissues were from fusiform gyrus because this area is implicated in autism [29-32]. The diagnosis of autism was confirmed using the Autism Diagnostic Interview-Revised [33] post-mortem through interviews with the parents and/or caregivers. Samples from subjects with known genetic causes of autism spectrum and related disorders (Rett, Asperger etc.) were excluded. Subject characteristics are shown in Tables 1 and 2.

**Table 1:** **Characteristics of autism tissue samples**

**Sample ID#**	**CASE #**	**Age (years)**	**Gender**	**PMI (hours)**	**Primary cause of death**	**ADI-R**	**Drug treatment history**
A1	AN01093	56	Male	19.48	Anoxic Encephalopathy	48	N/A
A2	UMB1174	7	Female	14	Seizure, Hypotension	44	Depakote, Dilantin, Tegretol
A3	AN00764	20	Male	23.7	Auto Trauma	50	Minocin
A4	AN08792	30	Male	20.3	GastroIntestinal Bleeding	41	Phenobarbital, Mysoline, Dilantin, Depakote, Cisapride, Clorazepate, Prolosec, Propulsid, Reglan, Tranxene
A5	AN06420	39	Male	13.95	Cardiac Tamponade	41	Synthroid, Depakote, Risperidol, Paxil, Blood pressure medication
A6	AN00493	27	Male	8.3	Drowning	-	Synthroid
A7	AN08873	5	Male	25.5	Asphyxia Due To Drowning	47	Prozac
A8	UMB797	9	Male	13	Drowning	50	Desipramine
A9	UMB1182	9	Female	24	Smoke Inhalation	-	N/A
A10	AN16641	9	Male	27	Seizure Disorder	46	Clonidine, Depakote, Dilantin, Lamictal, Ritalin, Tegretol
A11	AN16115	11	Female	12.88	Seizure & Drowning in Tub	44	Adderall, Dexadrine, Dilantin, Klonopin, Lamictal, Tegretol, Topomax
	Mean	**20.1**		**18.3**			
	SEM (+/−)	**4.9**		**1.8**			

**N/A** = Information Not Available; **PMI** = Post Mortem Interval; **ADI-R** = Autism Diagnostic Interview-Revised.

**Table 2:** **Characteristics of control tissue samples**

**Sample ID#**	**CASE #**	**Age (years)**	**Gender**	**PMI (hours)**	**Primary cause of death**	**Secondary cause of death**
C1	AN12552	56	Male	23.61	Traumatic asphyxia and crush injury	Cardiac Arrest
C2	AN17344	46	Male	25.9	Unknown	N/A
C3	AN14771	30	Male	23	Cardiac Arrhythmia	N/A
C4	UMB818	27	Male	10	Multiple injuries	Accident
C5	AN17425	16	Male	26.16	Unknown	N/A
C6	AN15240	36	Female	18.08	Unknown	N/A
C7	AN19760	28	Male	23.25	Unknown	N/A
C8	AN12240	51	Male	4.75	MI	N/A
C9	AN10606	56	Male	23	Myocardial infarction	N/A
C10	UMB1706	8	Female	20	Rejection of Cardiac Allograft Transplantation	N/A
C11	UMB1860	8	Male	5	Cardiac Arrhythmia	N/A
C12	UMB1407	9	Female	20	Asthma	N/A
C13	UMB1649	20	Male	22	Multiple injuries	N/A
	Mean	**28.5**		**18.8**		
	SEM (+/−)	**4.8**		**1.8**		

**N/A** = Information Not Available; **PMI** = Post Mortem Interval.

### Animal brain tissue samples

All experimental protocols were in compliance with the University of Calgary Conjoint Faculties Research Ethics Approval Board. Pregnant Wistar Han rats (The Jackson Laboratory, Bar Harbor, ME, USA or Charles River Laboratories, Wilmington, MA, USA) were ordered on embryonic day 3 (E3). The sodium salt of valproic acid (Sigma, St. Louis, MO, USA) was dissolved in 0.9% saline to achieve a concentration of 250 mg/ml. The dosing volume was 500 mg/kg. On E12.5, treated dams received a single intraperitoneal (i.p.) injection of 500 mg/kg VPA sodium salt, while control dams were injected with only saline [17,34]. Rats were born and reared in a quiet, temperature-controlled room and entrained to a 12-h light–dark cycle. Male pups from each group were sacrificed between P35-38. As the fusiform gyrus, which is part of the temporal and occipital lobes in humans, is not present in rodents, the lateral temporal neocortex was collected for analysis.

### RNA isolation and RT-PCR

Seven autism and eleven control samples from fusiform gyrus were used for qRT-PCR. RNA was isolated as described in reference [28]. Yield and purity of total cellular RNA were determined by absorbance at 260 and 280 nm, and RNA integrity was verified by agarose gel electrophoresis. cDNA was generated as previously described [28]. Samples containing double-distilled water in place of reverse transcriptase were included as negative controls (no-RT).

Real-time qPCR was performed as described [28]. Forward primer, 5′-GGC CCA GAT GCT GTC ATT AT-3′, and reverse primer, 5′-TTC TGC TCA GGA CAG AGG TT-3′, were used to detect full length TrkB (TrkB-FL), while truncated TrkB (TrkB-T1) was detected using forward primer, 5′-TGC CTT TTG GTA ATG CTG TTT-3′, and reverse primer, 5′-GGC TTC ATA TAG TAC AGC CTC CA-3′. Lastly, detection of the truncated isoform TrkB-Shc was performed using forward primer, 5′-GGC CCA GAT GCT GTC ATT AT-3′, and reverse primer, 5′-AGG CAT GGA TTT AGC CTC CT-3′. Copy numbers using absolute quantification and PCR efficiencies were calculated with MXPro Mx3000P Software (Stratagene). Only experiments in which the real-time PCR efficiency was between 90%-100% and standard curves yielded a R^2^ > 0.990 were used for analysis.

### Protein extraction from brain tissue

Rat tissues were homogenized in RIPA buffer (Thermo Scientific, Rockford, IL, USA) with protease (complete, Mini, EDTA-free) and phophatase (PhosSTOP) inhibitor cocktail tablets (Roche, Indianapolis, IN, USA). A similar homogenization buffer [35] was used for human samples. Homogenates were sonicated, incubated on ice for 15–30 min and then centrifuged at 13000 × g for 10–20 min at 4°C to precipitate insoluble debris. The supernatants were collected and protein concentrations were determined using the BCA protein assay kit (Thermo Scientific, Rockford, IL, USA) or the DC™ protein assay (Bio-Rad Laboratories, Mississauga, Ontario, Canada) as described by the manufacturer. Rat tissue lysates were pooled in order to handle a large number of samples that exceeded the lane capacity of a gel, while human homogenates were not pooled. Sample protein concentrations were adjusted to 4 μg/μl (rat) or 1.5-3.5 μg/μl (human) for Western blotting.

### Western blotting and densitometry

10 to 40 μg total protein (depending on the target) was separated on 8%-12% sodium dodecyl sulfate (SDS)-polyacrylamide gels or Mini-PROTEAN**®** Precast Gels (Bio-Rad, Hercules, CA, USA) under reducing conditions and transferred to polyvinylidene fluoride (PVDF) membranes (Bio-Rad, Hercules, CA, USA). Membranes were then blocked with a 1:1 solution of phosphate-buffered saline (PBS) pH7.4 and Odyssey Blocking Buffer (BB) (Cedarlane, Burlington, Ontario, Canada) or 5% nonfat milk in Tris-buffered saline solution containing 0.1% Tween 20 (TBS-T) for 1 hour. After blocking, the blots were probed overnight at 4°C with the following primary antibodies: TrkB (Cell Signaling Technology, Danvers, MA, USA, diluted 1:700), PI3K p85, Akt, phospho-Akt, mTOR, phospho-mTOR (Ser2448), 4E-BP1, phospho-4E-BP1, eIF4E, eIF4B, S6, phospho-S6, GFAP, βIII-Tubulin (Cell Signaling Technology, diluted 1:1000), p70S6K (Santa Cruz Biotechnology, diluted 1:500), and PSD-95 (Millipore, diluted 1:1000). Membranes were simultaneously probed with mouse monoclonal anti-β-actin antibody (Sigma, diluted 1:5000 or Cell Signaling Technology, diluted 1:1000) as a loading control.

After washing with TBS-T, rat blots were incubated with the secondary antibody horseradish peroxidase (HRP)-conjugated goat anti-rabbit (Bio-Rad, Hercules, CA, USA) for 1 hour at room temperature, washed with TBS-T, exposed to Hyper film ECL (Amersham Pharmacia Biotech, Carlsbad, CA, USA) and ultimately visualized using the enhanced chemiluminescence (ECL) detection system. Bands were detected and quantified using a ChemiDOC MP gel imaging system (Bio-Rad, Hercules, CA, USA).

After washing with PBS containing 0.5% Tween-20 (PBS-T), human tissue blots were incubated with the secondary antibodies IRDye 680-conjugated goat anti-rabbit and IRDye 800CW-conjugated goat anti-mouse (LI-COR Biosciences, Lincoln, NE, USA; diluted 1:8000) for 1 hour at room temperature, washed with PBS-T and scanned using an Odyssey® Infrared Imaging System (LI-COR Biosciences). Band intensities were quantified by densitometry with local background subtraction using LI-COR® Odyssey Software, version 2.0.

### Statistical analysis

Each Western blot for human tissue contained a standard curve consisting of different amounts of protein per lane (from 1 to 80 μg) from a single normal human cortex sample to allow normalization between blots and to ensure that the sample loading amount was in the linear range of detection for all targets tested.

For both RT-PCR and Western blot experiments, each human or rat sample was expressed as a ratio to its corresponding β-actin value.

Differences in the target mRNA or protein levels between autism, VPA and respective control samples were calculated by 2-tailed Student’s *t*-test with statistical significance set at p < 0.05.

## Results

### Decreased PI3K p85, Akt, mTOR, phospho-mTOR, p70S6K and eIF4B protein expression in idiopathic autism

**Figure 1:**
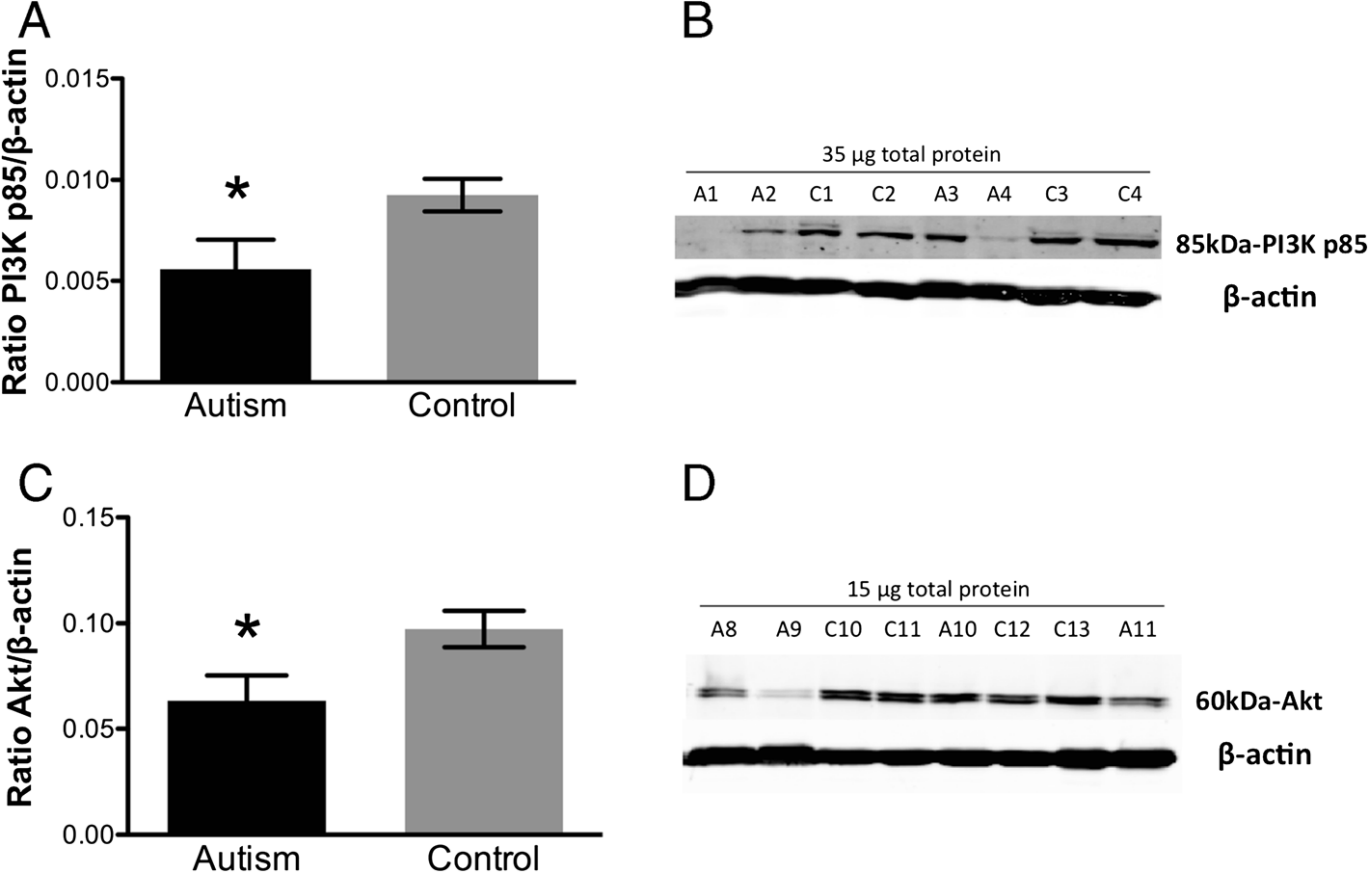
**Quantification of (A) PI3K p85 and (C) Akt protein expression in fusiform gyrus of autism and control samples by Western blotting.** Each sample was normalized to its β-actin. *p = 0.03 for PI3K p85 and Akt, 2-tailed *t* tests. Bars indicate mean ± SE. Autism, n = 11; control, n = 13. The mean from two independent Western blots per sample was used for statistical analysis. **(B)** and **(D)** Representative Western blots of fusiform gyrus showing autism (A) and control (C) cases. 35 μg for PI3K p85 and 15 μg for Akt of total protein from each autism and control sample were loaded.

Akt/mTOR pathway protein expression levels were examined in the fusiform gyrus of subjects with idiopathic autism versus control subjects. Fusiform gyrus is an area of the brain implicated in face discrimination and perception difficulties of autistic subjects [29,32]. Western blotting revealed a statistically significant decrease in PI3K p85 and Akt (*p = 0.03, Figure 1A,B; *p = 0.03, Figure 1C,D; 2-tailed *t* tests) protein in the fusiform gyrus of autistic subjects relative to controls.

**Figure 2:**
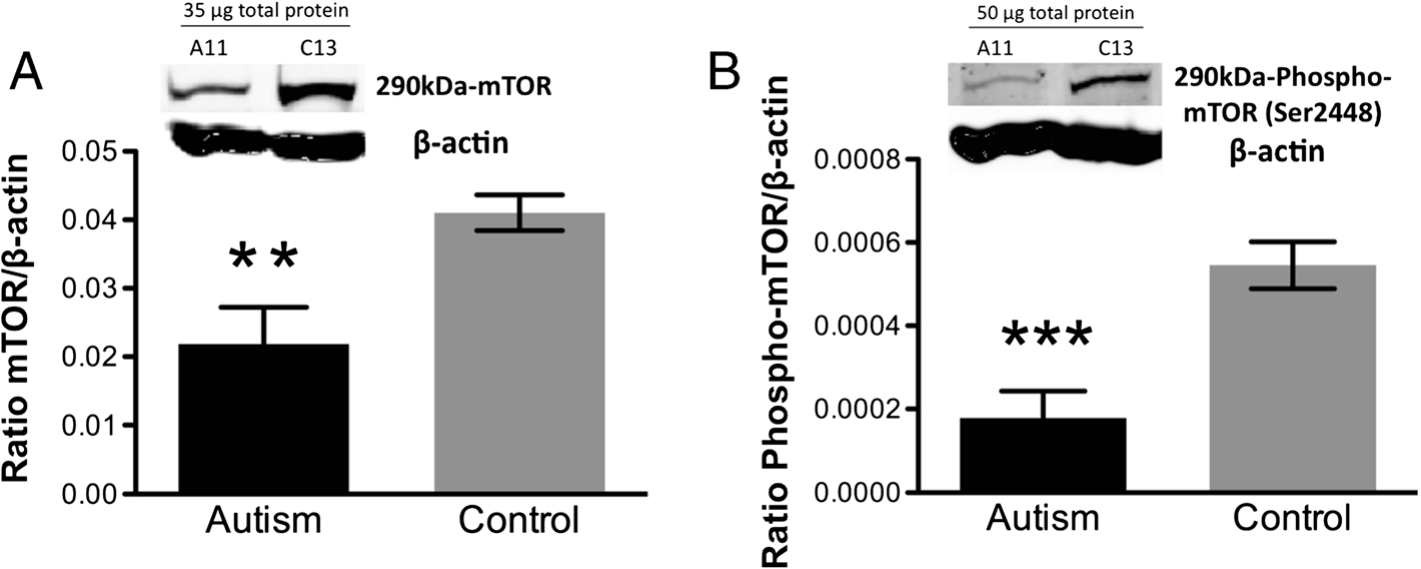
**Quantification by Western blotting and representative Western blots of (A) total mTOR and (B) phospho-mTOR protein expression in autism versus control fusiform gyrus samples.** Each sample was normalized to its β-actin. **p = 0.003 for mTOR, ***p = 0.0003 for phospho-mTOR, 2-tailed *t* tests. Bars indicate mean ± SE. Autism, n = 11; control, n = 13. The mean from two independent Western blots per sample was used for statistical analysis. 35 μg for mTOR and 50 μg for phospho-mTOR of total protein from each autism (A) and control (C) sample were loaded.

Total and phosphorylated mTOR were also decreased in autism versus control brain (**p = 0.003, Figure 2A; ***p = 0.0003, Figure 2B; 2-tailed *t* tests).

**Figure 3:**
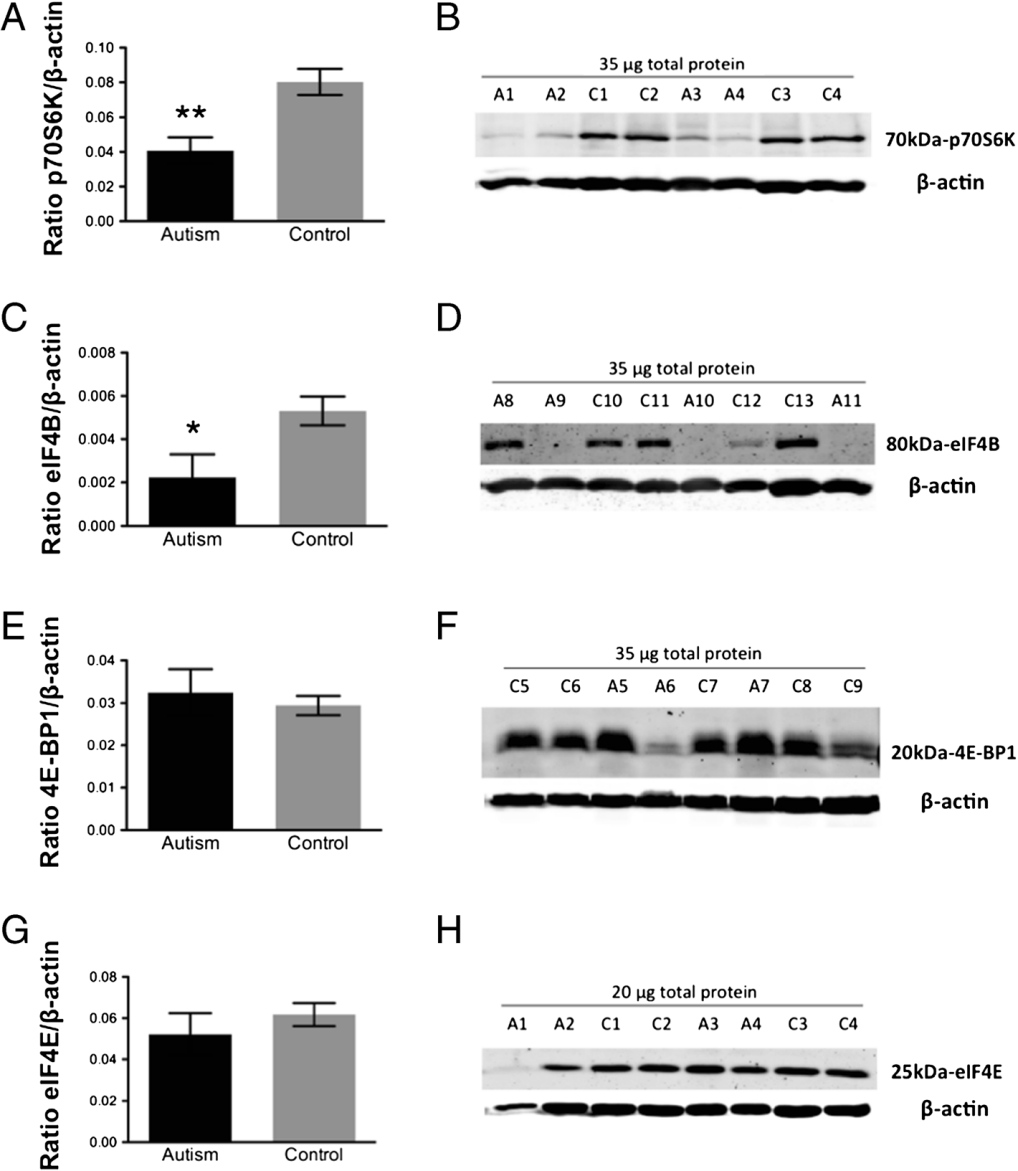
**Quantification of (A) p70S6K, (C) eIF4B, (E) 4E-BP1 and (G) eIF4E protein expression in fusiform gyrus of autism and control samples by Western blotting.** Each sample was normalized to its β-actin. **p = 0.002 for p70S6K, *p = 0.02 for eIF4B, p = 0.6 for 4E-BP1 and p = 0.4 for eIF4E, 2-tailed *t* tests. Bars indicate mean ± SE. Autism, n = 11; control, n = 13. The mean from two independent Western blots per sample was used for statistical analysis. **(B)**, **(D)**, **(F)** and **(H)** Representative Western blots of fusiform gyrus showing autism (A) and control (C) cases. 35 μg for p70S6K, eIF4B and 4E-BP1 and 20 μg for eIF4E of total protein from each autism and control sample were loaded.

We next examined the mTOR downstream effector pathways p70S6K/eIF4B and 4E-BP1/eIF4E involved in local dendritic protein translation. A statistically significant decrease in p70S6K and eIF4B protein was found (**p = 0.002, Figure 3A,B; *p = 0.02, Figure 3C,D; 2-tailed *t* tests) in the fusiform gyrus of autistic subjects relative to controls. However, there were no significant differences in protein expression levels of 4E-BP1 or eIF4E (p = 0.6, Figure 3E,F; p = 0.4, Figure 3G,H; 2-tailed *t* tests) in the fusiform gyrus of control *vs.* autism subjects.

### Reduced PSD-95 protein expression in idiopathic autism

**Figure 4:**
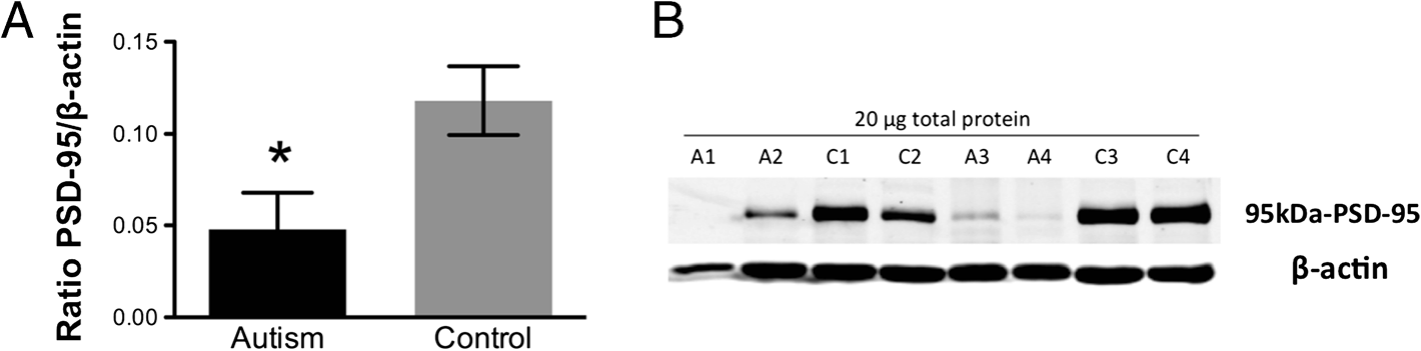
**Quantification of (A) PSD-95 protein expression in fusiform gyrus of autism and control samples by Western blotting.** Each sample was normalized to its β-actin. *p = 0.02, 2-tailed *t* test. Bars indicate mean ± SE. Autism, n = 11; control, n = 13. The mean from three independent Western blots per sample was used for statistical analysis. **(B)** Representative Western blots of fusiform gyrus showing autism (A) and control (C) cases. 20 μg of total protein from each autism and control sample were loaded.

Because the Akt/mTOR pathway can influence protein synthesis at spines, and de-regulated mTOR has been associated with spine deficits in monogenic disorders with high rates of autism [2,3,36], PSD-95 protein expression, a marker of excitatory synapses, was examined in the fusiform gyrus of autism versus control subjects. Western blotting revealed a significant decrease in PSD-95 protein levels (*p = 0.02, Figure 4A,B; 2-tailed *t* test) in individuals with idiopathic autism versus controls.

### Imbalance in TrkB protein isoforms in idiopathic autism

**Figure 5:**
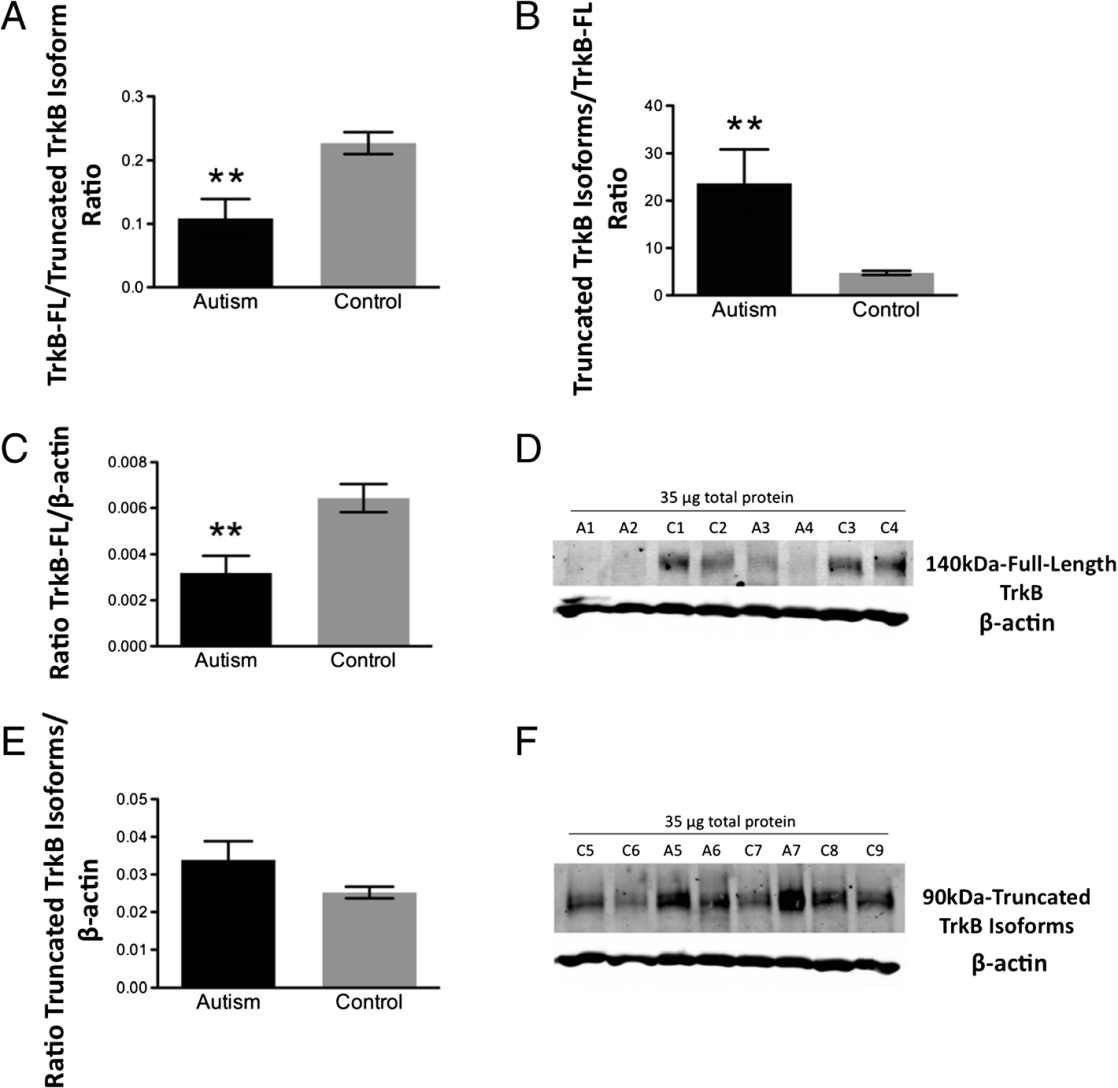
**Quantification of (A) full-length (FL)/truncated and (B) truncated/full-length (FL) TrkB isoform protein ratios in fusiform gyrus of autism and control samples.** **p = 0.002 and **p = 0.009, respectively, 2-tailed *t* tests. Bars indicate mean ± SE. Autism, n = 11; control, n = 13. **(C)** Quantification of full-length (FL) TrkB and **(E)** truncated TrkB isoform protein expression in fusiform gyrus of autism and control samples by Western blotting. Each sample was normalized to its β-actin. **p = 0.003 for TrkB-FL and p = 0.09 for truncated TrkB isoforms, 2-tailed *t* tests. Bars indicate mean ± SE. Autism, n = 11; control, n = 13. The mean from two independent Western blots per sample was used for statistical analysis. **(D)** and **(F)** Representative Western blots of fusiform gyrus showing autism (A) and control (C) cases. Lanes 2 to 5: standard curve consisting of different amounts of total protein from a single normal human cortex sample. Lanes 6 to 13: 35 μg of total protein from each autism and control sample.

**Figure 6:**
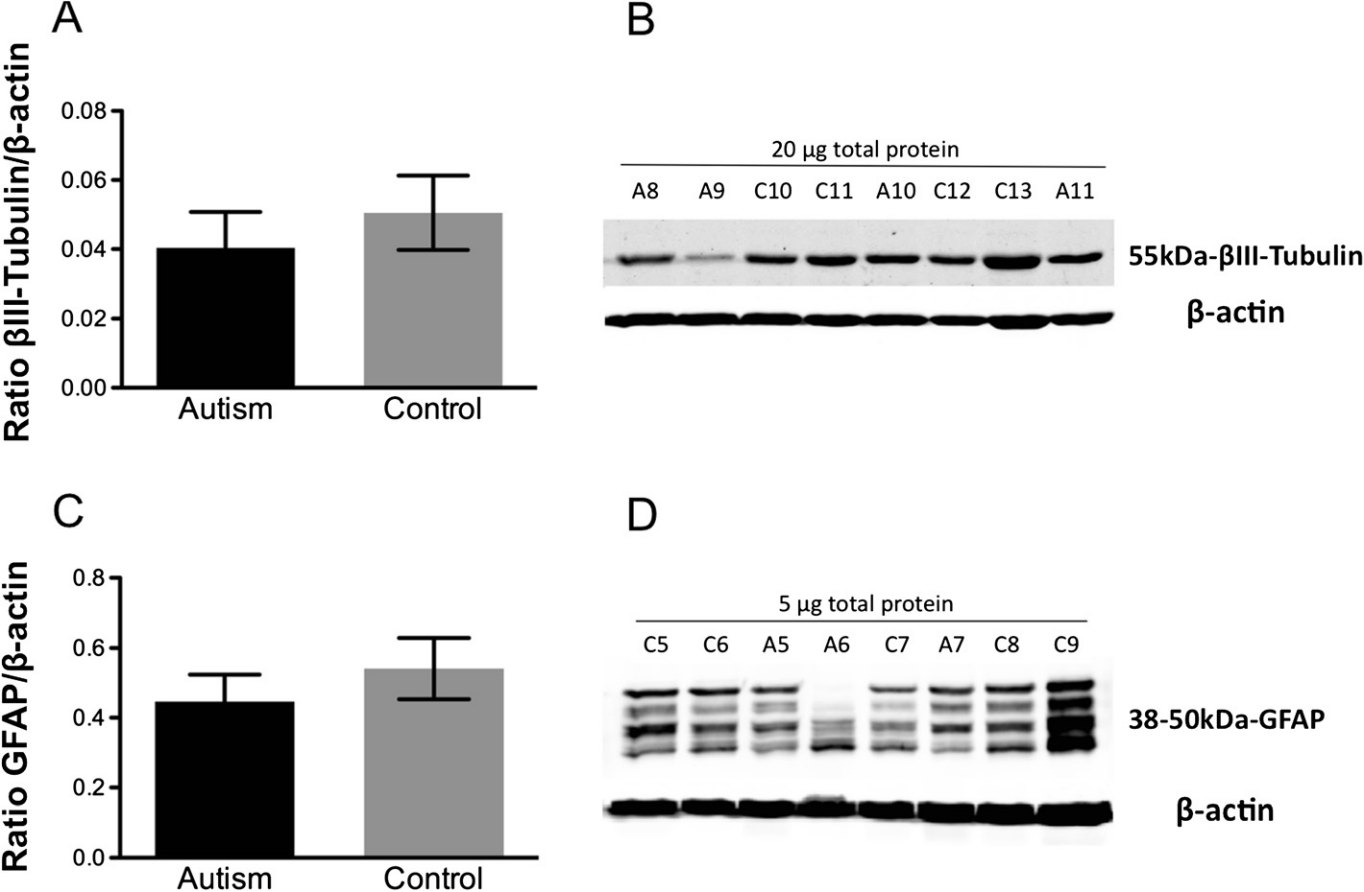
**Quantification of (A) Beta-III Tubulin and (C) GFAP protein expression in fusiform gyrus of autism and control samples by Western blotting.** Each sample was normalized to its β-actin. As reported by other studies [38,39], four bands ranging from 38 kDa to 50 kDa were detected for GFAP in all samples and quantified together. p = 0.5 for βIII-tubulin, p = 0.4 for GFAP, 2-tailed *t* test. Bars indicate mean ± SE. Autism, n = 11; control, n = 13. The mean from two independent Western blots per sample was used for statistical analysis. **(B)** and **(D)** Representative Western blots of fusiform gyrus showing autism (A) and control (C) cases. 20 μg for Beta-III Tubulin and 5 μg for GFAP of total protein from each autism and control sample were loaded.

The TrkB/Akt pathway is known to influence PSD-95 trafficking to spines [37]. Thus, differences in relative TrkB protein isoform levels in fusiform gyrus between autism and control groups were examined by Western blotting. A significant reduction in full-length TrkB (TrkB-FL)/truncated TrkB (TrkB-T1 + TrkB-Shc) isoform ratio and increase in the ratio of truncated TrkB isoforms/TrkB-FL (**p = 0.002, Figure 5A; **p = 0.009, Figure 5B; 2-tailed *t* tests) were observed in the fusiform gyrus of individuals with idiopathic autism versus controls. This may be due to significantly decreased TrkB-FL (**p = 0.003, Figure 5C,D; 2-tailed *t* test) as well as a trend towards increased truncated TrkB (p = 0.09, Figure 5E,F; 2-tailed *t* test). TrkB-FL and truncated TrkB are differentially expressed in different cell types: TrkB-FL is neuronal whereas truncated TrkB isoforms are widely expressed in glia. Therefore, we measured protein levels of GFAP (glial) and βIII-Tubulin (neuronal) markers. There were no significant differences in GFAP or βIII-Tubulin protein expression in the fusiform gyrus (p = 0.4, Figure 6A,B; p = 0.5, Figure 6C,D; 2-tailed *t* tests) between control and autism subjects, which precludes differential cell loss as a mechanism for TrkB isoform alterations. There were also no differences between groups in β-actin levels (p = 0.7, 2-tailed *t* test, data not shown), which were used to normalize samples on the Western blots.

No significant differences in TrkB isoform mRNA levels were found in the fusiform gyrus between control and autism subjects (TrkB-FL: p = 0.1; TrkB-T1: p = 0.3; TrkB-Shc: p = 0.7; 2-tailed *t* tests; data not shown).

### Decreased mTOR signaling in VPA-exposed rats

In this study, we specifically chose to examine idiopathic autism. As epigenetics are thought to play a key role in causing idiopathic autism, we also examined the brains of rats exposed to the HDAC inhibitor VPA *in utero*. We measured Akt, mTOR, S6K, and 4E-BP1 total and phosphorylated protein levels in VPA- versus saline-exposed rat lateral temporal neocortices.

**Figure 7:**
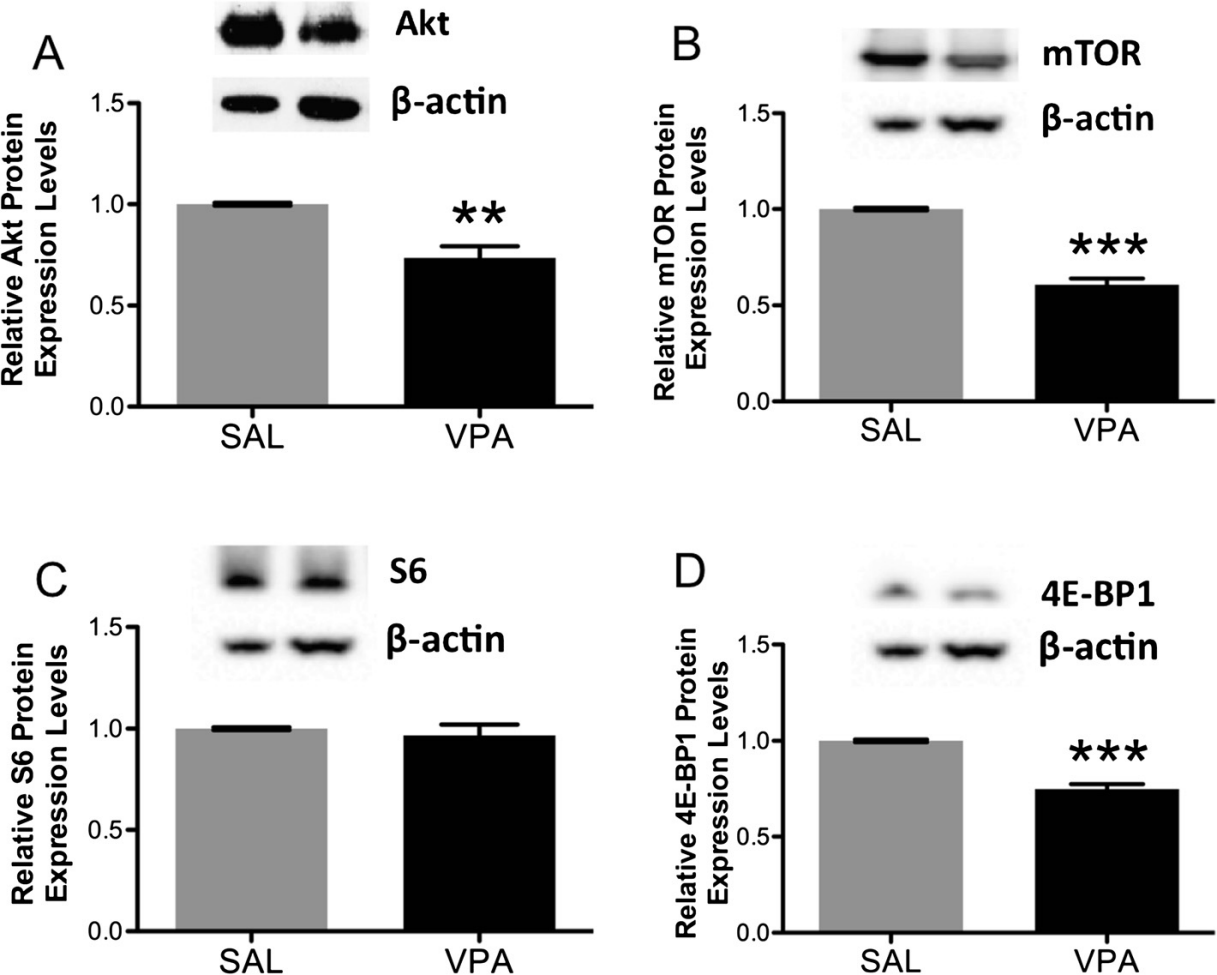
**Quantification by Western blotting and representative Western blots of total (A) Akt, (B) mTOR, (C) S6 and (D) 4E-BP1 protein expression in VPA- versus saline (SAL)-exposed rat lateral temporal neocortices.** Each sample was normalized to its β-actin, and VPA-exposed values were expressed relative to SAL-exposed. All p-values were calculated by 2-tailed *t* test. **Akt**: **p = 0.002, SAL, n = 5; VPA, n = 5; **mTOR**: ***p < 0.0001, SAL, n = 8; VPA, n = 8; **S6**: p = 0.5, SAL, n = 8; VPA, n = 8; **4E-BP1**: ***p < 0.0001, SAL, n = 6; VPA, n = 6. Bars indicate mean ± SE. The mean from three independent Western blots per sample was used for statistical analysis.

**Figure 8:**
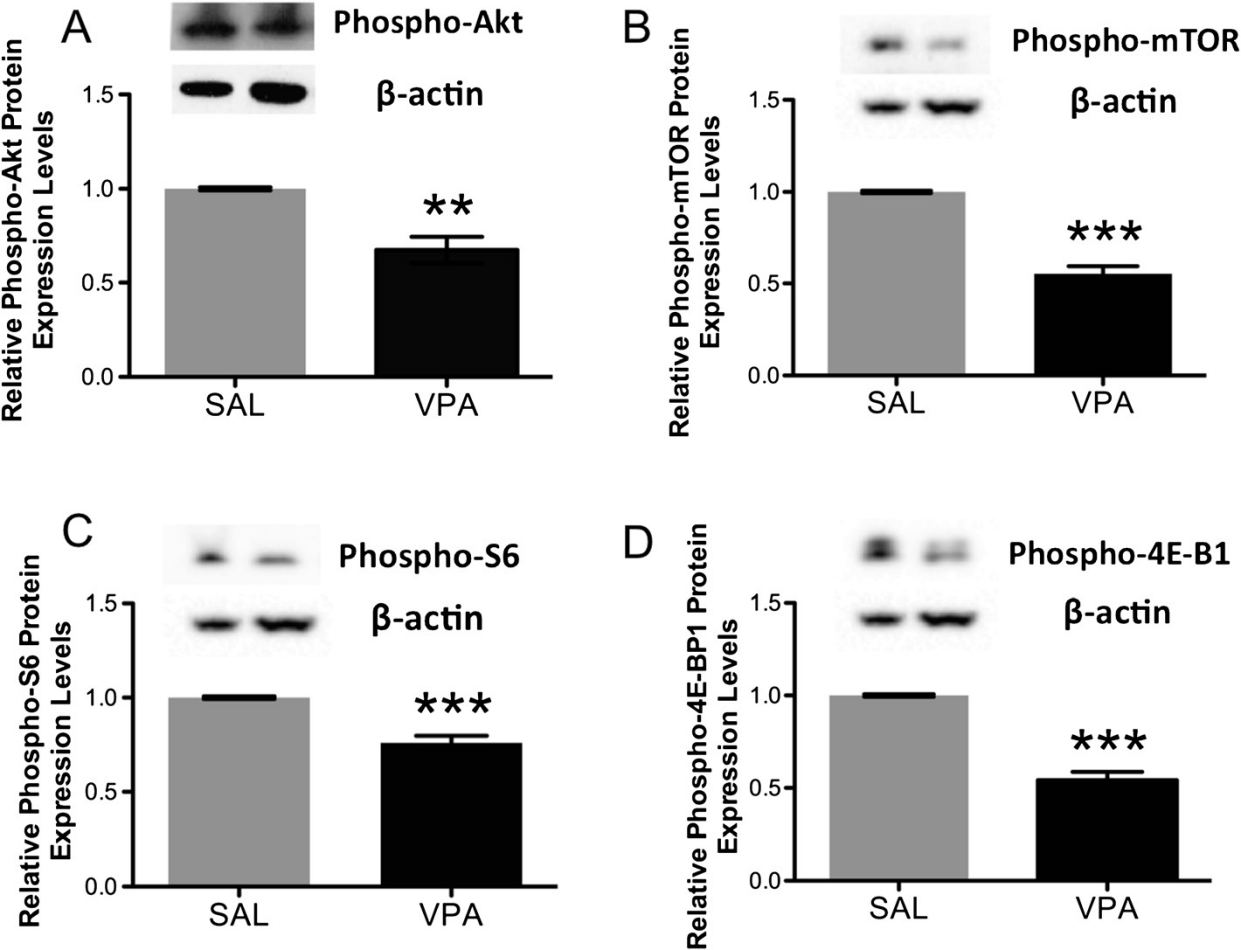
**Quantification by Western blotting and representative Western blots of phosphorylated (A) Akt, (B) mTOR, (C) S6 and (D) 4E-BP1 protein expression in VPA- versus saline (SAL)-exposed rat lateral temporal neocortices.** Each sample was normalized to its β-actin, and VPA-exposed values were expressed relative to SAL-exposed. All p-values were calculated by 2-tailed *t* test. **Phospho-Akt**: **p = 0.004, SAL, n = 4; VPA, n = 4; **phospho-mTOR**: ***p < 0.0001, SAL, n = 8; VPA, n = 8; **phospho-S6**: ***p < 0.0001, SAL, n = 8; VPA, n = 8; **phospho-4E-BP1**: ***p < 0.0001, SAL, n = 6; VPA, n = 6. Bars indicate mean ± SE. The mean from three independent Western blots per sample was used for statistical analysis.

Western blotting revealed a statistically significant decrease in both total and phosphorylated Akt (**p = 0.002, Figure 7A; **p = 0.004, Figure 8A; 2-tailed *t* tests), mTOR (***p < 0.0001, Figure 7B; ***p < 0.0001, Figure 8B; 2-tailed *t* tests) and 4E-BP1 (***p < 0.0001, Figure 7D; ***p < 0.0001, Figure 8D; 2-tailed *t* tests) in VPA-exposed rats compared to saline-exposed controls. However, only phosphorylated (***p < 0.0001, Figure 8C; 2-tailed *t* test) but not total (p = 0.6, Figure 7C; 2-tailed *t* test) S6 protein expression was significantly reduced in VPA- versus saline-exposed rats.

## Discussion

Studies of single-gene disorders with a high prevalence of autism such as Rett syndrome (mutations in MeCP2), fragile X syndrome (mutations in FMR1), tuberous sclerosis (mutations in TSC1/TSC2), neurofibromatosis (mutations in NF1), and macrocephaly (mutations in PTEN), point to the Akt/mTOR pathway as a good candidate for involvement in autism pathogenesis [2-5,36,40,41]. In the present study, we demonstrate that, in contrast to fragile X syndrome, tuberous sclerosis, neurofibromatosis 1 and macrocephaly which exhibit increased mTOR pathway components [2,3,5], the mTOR pathway is decreased in idiopathic autism. Our findings are consistent with previous reports of reduced mTOR pathway in Rett syndrome [36] and decreased Akt total protein and phosphorylation in frontal cerebral cortex from autistic patients [42]. This supports the idea that mTOR pathway deviations in either direction can adversely affect establishment, maintenance and function of neural networks and thus ultimately cause autism’s cognitive and behavioural deficits [43].

As several of our subjects with autism exhibited seizures (n = 4), while others did not (n = 6), results from these two groups of patients were compared. No differences in levels of any of the proteins studied (PI3K p85, Akt, total and phosphorylated mTOR, TrkB isoforms, p70S6K, eIF4B or PSD-95) were found between autism cases with seizure disorder and those without (p > 0.05, 2-tailed *t* tests). This is consistent with our previous findings for BDNF [28]. BDNF mRNA is highly sensitive to seizures, and yet in that study, none of our autistic subjects with seizures (the same ones as used in this study) exhibited higher BDNF mRNA levels than those without.

mTOR regulates protein synthesis at synapses via two distinct downstream pathways which are responsible for promoting translation of different pools of mRNAs [44,45]. The p70S6K and eukaryotic initiation factor 4B (eIF4B) cascade regulates translation of mRNAs encoding translational machinery components such as elongation and initiation factors and ribosomal proteins, while the eukaryotic initiation factor 4E-binding protein 1 (4E-BP1) and eukaryotic initiation factor 4E (eIF4E) pathway control translation of 5′ capped mRNAs coding for structural and functional synaptic proteins [46,47]. Single nucleotide insertions in the promoter of the mTOR effector eIF4E are found in individuals with autism from two unrelated families [5], and linkage of autism to the eIF4E region on chromosome 4q shown in genome-wide association studies [48] suggest that this pathway may be preferentially affected in genetic causes of autism. Conversely, in our study, autistic subjects had significantly decreased p70S6K and eIF4B, while there were no changes in 4E-BP1 or eIF4E in these patients, pointing to specific deficits in mTOR-dependent translation via the p70S6K/S6 pathway in idiopathic autism.

Overall, nine of our eleven autism subjects, regardless of whether they had concurrent seizure disorder, exhibited decreases throughout the mTOR pathway (Table 3). The direction of change is uniform for each subject, suggesting coordinated or feedback regulation of the pathway. Our findings demonstrate that disruptions in mTOR and its signaling cascade components are widespread in the autism population and not limited to genetic forms of autism.

**Table 3:** **Distribution of analyzed protein targets from autism subjects relative to controls**

	**Autism patients with no seizure disorder**							**Autism patients with seizure disorder**			
	**AN01093 (A1)**	**AN00764 (A3)**	**AN00493 (A6)**	**UMB1182 (A9)**	**AN06420 (A5)**	**UMB797 (A8)**	**AN08873 (A7)**	**UMB1174 (A2)**	**AN08792 (A4)**	**AN16641 (A10)**	**AN16615 (A11)**
**TrkB-FL**	***−2.0 SD***	***−1.5 SD***	***−2.0 SD***	***−1.5 SD***	0.5 SD	***−0.5 SD***	0.5 SD	***−2.0 SD***	***−2.0 SD***	***−1.5 SD***	***−1.5 SD***
**PI3K p85**	***−2.0 SD***	***−1.5 SD***	***−2.0 SD***	***−2.0 SD***	0.5 SD	***−0.5 SD***	0.5 SD	***−2.0 SD***	***−2.0 SD***	1.5 SD	***−1.5 SD***
**Akt**	***−2.0 SD***	***−0.5 SD***	***−1.5 SD***	***−2.0 SD***	1.5 SD	***−0.5 SD***	0.5 SD	***−1.5 SD***	***−1.5 SD***	***−0.5 SD***	***−1.5 SD***
**mTOR**	***−2.0 SD***	***−2.0 SD***	***−2.0 SD***	***−2.0 SD***	1.5 SD	***−0.5 SD***	0.5 SD	***−2.0 SD***	***−2.0 SD***	***−1.5 SD***	***−2.0 SD***
**Phospho-mTOR**	***−1.5 SD***	***−2.0 SD***	***−2.0 SD***	***−2.0 SD***	0.5 SD	***−0.5 SD***	***−0.5 SD***	***−2.0 SD***	***−2.0 SD***	***−2.0 SD***	***−2.0 SD***
**p70 S6** ** Kinase**	***−2.0 SD***	***−1.5 SD***	***−1.5 SD***	***−2.0 SD***	0.5 SD	***−0.5 SD***	***−0.5 SD***	***−1.5 SD***	***−2.0 SD***	***−1.5 SD***	***−1.5 SD***
**eIF4B**	***−2.0 SD***	***−2.0 SD***	***−2.0 SD***	***−1.5 SD***	1.5 SD	2.0 SD	***−0.5 SD***	***−1.5 SD***	***−2.0 SD***	***−2.0 SD***	***−2.0 SD***
**PSD-95**	***−1.5 SD***	***−1.5 SD***	***−1.5 SD***	***−1.5 SD***	0.5 SD	0.5 SD	0.5 SD	***−1.5 SD***	***−1.5 SD***	***−1.5 SD***	***−1.5 SD***

**SD** = Standard deviation. The mean of each target for the control group was used as a reference to calculate the corresponding standard deviation values for each target. **Bold-Italics** = 0.5-2.0 standard deviations less than the mean of controls. **Not Bold** = 0.5-2.0 standard deviations greater than the mean of controls.

In support of this evidence, our animal data point to epigenetic mechanisms as a likely underlying cause of these disruptions. We are the first to report that maternal exposure to the anticonvulsant VPA, an HDAC inhibitor, results in decreased mTOR signaling in lateral temporal neocortices of rats. Significantly down-regulated protein levels of mTOR as well as its upstream/downstream effectors were associated with a significant reduction in the overall amount of social play behaviors and an attenuated response to playful attacks in VPA- versus saline-exposed control rats [34,49], corroborating the hypothesis that reduced mTOR pathway signaling contributes to autistic behaviors.

In contrast to idiopathic autism which exhibits only S6 pathway deficits, both mTOR downstream effector cascades appear to be affected in VPA-exposed rats, as deficits were found in phosphorylated S6 and 4E-BP1. It is possible that these differences in down-regulated mTOR effectors between rat and human may specifically underlie autistic-like behavior caused by VPA treatment.

A consequence of dysfunctional mTOR signaling is deficits in spine protein translation and changes in spine morphology, density and dynamics [3,36]. Abnormal spine density and morphology have been reported in cortical neurons from autistic subjects [7,50]. Interestingly, we observed that decreased mTOR was associated with a significant reduction of PSD-95, a key postsynaptic density organizing molecule, in autistic subjects compared to controls. There are several mechanisms at work which might impact levels of PSD-95. The high-affinity brain-derived neurotrophic factor (BDNF) receptor TrkB regulates trafficking of PSD-95 at synapses via PI3K-Akt [37]. Interestingly, TrkB involvement in autism pathogenesis is substantiated by the finding of an association between *trkB (nTrk2)* gene variants and autism [51]. Consistently, we discovered significantly decreased protein levels of full-length TrkB (TrkB-FL), a trend towards increased truncated TrkB isoform protein levels and significantly altered TrkB isoform ratios in autism versus control subjects, supporting a TrkB isoform imbalance in autism similar to other neuropsychiatric disorders such as schizophrenia [52,53]. By interfering with PSD-95 transport at synapses via PI3K/Akt, decreased TrkB-FL may negatively affect spines and thus might impair neural networks which subserve higher cognitive functions and behavior. Reduced TrkB might also contribute to the Akt/mTOR pathway down-regulation seen in idiopathic autism, since TrkB-FL contains the intracellular catalytic tyrosine kinase domain needed to activate TrkB-mediated signaling cascades, including the Akt/mTOR pathway, upon BDNF binding [54]. Additionally, truncated TrkB isoforms, which lack the tyrosine kinase activity and thus can only bind and sequester BDNF but cannot activate downstream signaling cascades [55,56], could act as negative regulators by trapping BDNF or by forming heterodimers with TrkB-FL receptors [57-59], and further down-regulate the Akt/mTOR pathway.

TrkB-FL is expressed almost exclusively in pyramidal neurons and interneurons [60-62], whereas truncated isoforms are found in both neurons and glia [63,64]. However, no differences in either GFAP or βIII-Tubulin levels between autism and control subjects were detected, demonstrating that these TrkB isoform imbalances are not due to a shift in the proportion of neurons versus glia. Also, as no alterations were observed in TrkB isoform mRNA, it is likely that translational and/or post-translational mechanisms may contribute to the abnormal ratio of TrkB isoforms seen in the autistic brain.

In summary, our study is the first to demonstrate major decreases in protein expression and phosphorylation for mTOR and components of its downstream signaling pathways in subjects with idiopathic autism. These data demonstrate that deficits in this pathway are widespread in idiopathic autism and that up- or down-regulation of this pathway can have equally disruptive consequences. Notably, mTOR pathway deficits are supported by similar alterations in VPA-exposed rats, pointing to epigenetic mechanisms as the underlying molecular substrate of these disruptions. Despite minor differences in down-regulated mTOR cascade effectors, our human and animal data identify dysfunctional mTOR as a common molecular mechanism underlying autistic traits and suggest that a disruption of this pathway in either direction can have adverse consequences for dendritic spines, as supported by decreased PSD-95, and thus ultimately for synaptic development and function. In conclusion, our present findings highlight that decreased mTOR is likely to adversely affect spines and ultimately cortical circuits implicated in higher cognitive functions and behavior causing autistic phenotypes.
